# On the Use of Opium in Inflammation

**Published:** 1847-09

**Authors:** 


*On the Use of Opium in Inflammation.* By W. H. Ranking, M. D., late Physician to the Suffolk General Hospital. The legitimate sphere of action of *opium*, in the treatment of inflammatory diseases is, we conceive, a point upon which our notions have arrived at tolerable precision. Under whatever modifications of individual circumstances attending such diseases the beneficial action of opium is observed, one well-marked morbid condition has, according to my observation, existed in every case, and that is an excitement of the nervous system, altogether disproportionate to the exaggeration of vascular action. This excitement is not shown in the existence of spontaneous pain alone, as we know that that symptom may be insignificant, or altogether absent, in instances of the most extensive and destructive inflammation; neither is it shown mainly by increased sensibility to local impressions. The excitement to which I allude, exhibits in disorders of the sensory and motor functions of the nervous system chiefly, and consists in watchfulness, or transient delirium, irregular respiration, and especially in restlessness and jactitation. In this condition of things, whatever be the violence of the local inflammation, or whatever organ be affected (excepting the brain in some instances,) opium is imperatively called for. In other words, whenever, during the existence of inflammation, symptoms indicative of a loss of balance between the nervous and vascular systems exhibit themselves, sedative medicines are demanded in doses proportionate to the nervous preponderance.

This want of balance declares itself, I believe, chiefly under two conditions—1st, the existence of inflammation in a constitution naturally excitable, or in which the general powers have been reduced by the disease itself, by treatment, or by contingent circumstances relating to food, air, &c.; and, 2d, in inflammation of organs or tissues, the implication of which, induces a state of things more or less approaching to that condition which, for want of a better term, we are in the habit of calling *shock*. In illustration of the first division, we may mention inflammation occurring in the hysterical constitution. In these cases, the phenomena which depend upon irritation of the nervous centres, take so decided a lead in the symptomatology of the case, that until they are controlled by opium, or some, under certain circumstances, more appropriate sedative, the inflammatory symptoms proper do not display themselves with their characteristic features. Again, inflammation may attack an ill-fed or previously debilitated individual; or the inflammation may have been too actively combated by bloodletting, mercury, &c., without reference to the deficient resiliency of constitution, which, in children, more particularly, may lurk behind an appearance ostensibly robust. In these cases there may exist from the first, or there comes on assuredly at no distant period, a condition in which opium becomes necessary to save life to prevent, in fact, in the latter case, the anomaly of the patient "dying cured."

Under the second class of cases in which opium becomes a necessary part of the treatment, or is even mainly to be relied on, is inflammation of an organ or tissue largely supplied with ganglionic nerves, and in which, for this reason, the nervous system requires a large share of attention in the treatment of the case. Such is peritonitis or enteritis, either idiopathic or secondary; such are, also, one form of delirium tremens, diffuse cellular inflammation, and more particularly, phlebitis, the inner membrane of veins having the closest analogy to serous membrane in many respects, but especially in its large supply of organic nerves. In all these inflammations, the usual battery of antiphlogistics is worse than useless, unless combined with the liberal exhibition of opium.

The symptoms either existing *ab initio*, or, as is more commonly the case, coming on in the course of the disease, which indicate the necessity for opium, can only become familiar to the practitioner by clinical observation; but as far as written descriptions can be relied upon, it may be stated, that the broad expression of this condition consists in a failure in the power or regularity of the pulse, pallor of the countenance, moist skin, (but not in all cases,) tendency to incoherence, with restlessness, sleeplessness, and, in an aggravated form, jactitation. This is the broad outline, so to speak, of the state referred to, but it declares itself in minor degrees, with which experience alone can render us familiar, and the appreciation of which is in itself sufficient, in many cases, to make the difference between a successful and an unsuccessful practitioner; for to persevere in antiphlogistic treatment, or to withhold opium, when these indications offer themselves, is to destroy the patient.

In the exhibition of opium when these symptoms show themselves in inflammation, I know of no drawback,—no contraindication which should weigh for one moment against its paramount necessity. Be the skin sweating or dry, the tongue moist or dry, the bowels constipated or not, opium must be given. The constipated bowels, which are regarded by some as inducing the necessity for hesitation in the use of the medicine, I look upon as of the least importance in the generality of inflammations; in some, as in enteritis, a quiescent state of the bowels is even needful; and were it not so, the probability is, that if the case has been properly managed at first, such a clearance will have been effected as will render any risk from accumulation comparatively small.

[On the same subject Dr. Durrant observes:]

"The treatment of internal inflammation by opium," is an important practical subject, and one which I trust that we shall sooner or later see discussed in the pages of the *Provincial Journal*, by not a few only of its many talented contributors. Without entering upon the theory of the action which opium exerts upon healthy and diseased organs, I can most fully assent to the opinions which have been recently expressed by Drs. Chambers and Ranking, in reference to its utility in the treatment of many cases of acute internal inflammation. The power of allaying pain, subduing nervous excitability, and thereby reducing the irritability of the heart's action, peculiarly belongs to this drug; and however difficult or impossible it may be to instance, by writing, those particular conditions in which its adoption is so generally followed by benefit; still, how vividly will the reflecting physician, unshackled by hypothetical notions, revert to the satisfactory results which in his hands have attended its use.

In the selection of the cases most requiring the opium treatment, as also the precise period at which this medicine is called for, (and this is all important,) medical tact and observant experience can alone direct. A volume upon the subject would, in many instances, utterly fail to indicate the peculiar circumstances requiring opium, but in which to give it is recovery to the patient,—to withhold it is probable destruction; and who cannot call to mind the look of gratitude beaming in the countenance of the sufferer relieved, if not rendered out of danger, by the timely and judicious exhibition of this valuable drug? Independently of those diseases in the treatment of which opium is commonly prescribed, as delirium tremens, and some other nervous affections, there exist others, in which at one period or other of their course, the exhibition of opiates is attended with the happiest results.

In the second stage of pneumonia, if the cough be constant and harrassing, the expectoration scanty, and the countenance anxious, with an exalted state of nervous sensibility, full doses of opium at bedtime often act like a charm. In pleurisy, prior to effusion, the same medicine administered at night, giving mercury *per se* during the day, is equally valuable. If effusion obtain, the balance of the circulation is often so much interfered with, that the propriety of opium, in these cases, becomes questionable. In rheumatism the best effects result from opiates; but in this disease, as in pleurisy, I believe that more benefit will be derived from administering opium in full doses at night, with mercury during the day, instead of the more common practice of combining the the two remedies. In croup, also, in lymphatic children, after the urgency of the symptoms has abated, opium to prevent spasm is often imperatively called for. In peritonitis, enteritis, diarrhœa, and dysentery, opium judiciously used is invaluable.

The forms which I myself prefer are the crude opium and the muriate of morphia.

In reference to the constipating effects of this drug upon the bowels, I do not regard it as any reason for withholding its use. In enteritis and peritonitis, it becomes often of paramount importance, while allaying pain, to insure quietude in the intestinal tract; and not unfrequently shall we find obstinate constipation attended with, and perhaps produced by, spasm, effectually relieved by the free administration of opium. Within the last few weeks, I have witnessed the most decidedly beneficial result obtain from the exhibition of opium, in strangulated irreducible hernia, for the relief of which neither the patient (a female, aged 64) nor her friends would sanction an operation. After all other measures had been adopted by the surgeon in attendance, without avail, and repeated stercoraceous vomiting taking place, a grain of solid opium was administered every two hours, with the effect of not only allaying the vomiting, but also (after half a drachm had been taken) of inducing free and repeated evacuation of the bowels, which function had not previously been performed for fourteen days. The patient, although relieved from the consequences of the hernia, ultimately sank from exhaustion, the friends pertinaciously refusing a *post-mortem* examination.

In thus briefly advocating the powers of opium in the treatment of internal inflammation, I must again repeat, that it is clinical observation alone that will suffice to demonstrate, in many instances, the particular time and circumstances by which its exhibition must be regulated. When calculated to benefit, the relief which it affords is often immense, while, on the contrary, if given under disadvantageous conditions, its injurious effects may be irreparable. —*Ranking's Abstract.*

